# The Ratio of CD86+/CD163+ Macrophages Predicts Postoperative Recurrence in Stage II-III Colorectal Cancer

**DOI:** 10.3389/fimmu.2021.724429

**Published:** 2021-08-26

**Authors:** Guozeng Xu, Lei Jiang, Cheng Ye, Guizhen Qin, Zhanxiong Luo, Yuzhen Mo, Jian Chen

**Affiliations:** ^1^Department of Oncology, Liuzhou People Hospital, Guangxi Medical University, Liuzhou, China; ^2^Department of Pathology, Yantai Yuhuangding Hospital, Qingdao University, Yantai, China; ^3^Department of Pathology, Liuzhou People Hospital, Guangxi Medical University, Liuzhou, China; ^4^Department of Radiation Oncology, Guangzhou Red Cross Hospital, Jinan University, Guangzhou, China; ^5^Department of Medical Oncology, Yantai Yuhuangding Hospital, Qingdao University, Yantai, China

**Keywords:** CD86, CD163, ratio, recurrence, colorectal cancer, macrophage

## Abstract

Tumor-associated macrophages (TAMs) are pivotal for tumor progression and metastasis. We investigated the stromal CD86+TAM/CD163+TAM (CD86/CD163) ratio as a novel prognostic biomarker for stage II-III colorectal cancer (CRC). Two independently clinical cohorts of stage II-III CRC were retrospectively enrolled in this study. TAMs were detected using immunohistochemical staining for CD86 and CD163. The stromal CD86/CD163 ratio was calculated as a prognostic biomarker for recurrence-free survival (RFS) and overall survival (OS). Patients with a low CD86/CD163 ratio had shorter RFS (HR=0.193, *p*<0.001) and OS (HR=0.180, *p*<0.001) than patients with a high CD86/CD163 ratio in the training cohort. CD86/CD163 ratio may be an independent predictor for RFS (HR=0.233, *p*<0.001) and OS (HR=0.224, *p*<0.001) in the training cohort. We obtained equivalent results in the validation cohort. The CD86/CD163 ratio tends to have better predictive values than tumor stage in the training (AUC: 0.682 *vs* 0.654, *p*=0.538) and validation (AUC: 0.697 *vs* 0.659, *p*=0.586) cohorts. CD86/CD163 ratio effectively predicts RFS for stage II (HR=0.203, *p*<0.001) and stage III CRC (HR=0.302, *p*<0.001). CD86/CD163 ratio also effectively predicts RFS in CRC patients with adjutant chemotherapy (HR=0.258, *p*<0.001) and without adjutant chemotherapy (HR=0.205, *p*<0.001). The stromal CD86/CD163 ratio could be used for individual risk assessment of recurrence and mortality for stage II-III CRC. Together with tumor stage, this ratio will aid in the personal treatment.

## Introduction

Colorectal cancer (CRC) is one of the most commonly diagnosed tumors worldwide with about 0.78 million cancer-related deaths every year (1, 2). Approximately 60% of CRC patients were diagnosed with stage II and III disease at diagnosis (3–5). Despite remarkable improvement in new therapeutic techniques and strategies in the past two decades, postoperative recurrence occurs in approximately 30% of these patients (3–5). Furthermore, the benefit of adjutant chemotherapy is only noted in 5% of stage II and 15-20% of stage III patients (6–10). A significant proportion of CRC patients will experience adverse effects of chemotherapeutic drugs (3–10). These results highlight the unmet need to develop novel powerful prognostic biomarkers that allow for better recurrence stratification and optimal treatment strategies.

Accumulating evidence demonstrate that the immune microenvironment significantly affects tumor progression and metastasis (11).The immune microenvironment may provide abundant resources for identifying novel recurrence biomarkers (11). Macrophage is a main cellular subtype in the immune microenvironment and is commonly known as tumor-associated macrophages (TAMs) (12). Different macrophage subtypes have distinct functions in tumor progression. Generally, TAMs are separated into two distinct polarized states, including classically (M1), or alternatively (M2) activated macrophage (13–16). Classically activated (M1) TAMs, which express increased levels of iNOS, CD86, and CD169, play resistant roles in tumor progression and metastasis. In contrast, alternatively activated (M2) TAMs express increased levels of CD163, CD206, and CD204 (17–19). M2 TAMs in tumor tissues play a vital role in the suppression of tumor-associated immune responses and the enhancement of tumor invasion and metastasis (15–19). It should be noted that TAMs may be highly plastic cells and consist of a spectrum of activated states. And with M1-type and M2-type macrophages may only represent the extremes on each opposing end (18–20). We hypothesized that low CD86+ and high CD163+ TAM levels were clearly correlated with aggressive tumor phenotypes. Although the biology mechanism behind CD86/CD163 ratio is well known, the use of this ratio hasn’t been assessed for predicting tumor recurrence in CRC.

In this study, we evaluated the infiltration of macrophages marked with CD86 and CD163 in tumor tissues by the immunohistochemistry technique and focused on CD86/CD163 ratio, according to the STROBE guidelines (21). We aimed to provide enough evidence for the recurrence-risk stratification of stage II-III CRC.

## Materials and Methods

### Study Design and Participants

Two independently clinical cohorts of stage II-III CRC were retrospectively enrolled at two different medical centers. The training cohort consisted of 310 CRC patients from Yantai Yuhuangding Hospital between January 1, 2012 and December 31, 2015 to define the optimal cutoff point of the stromal CD86/CD163 ratio and to determine the prognostic efficacy of CD86/CD163 ratio, with the median follow-up time of 57.5 months. The validation cohort consisted of 139 CRC patients from Guangzhou Red Cross Hospital between January 1, 2013 and December 31, 2015 to validate the prognostic efficacy of CD86/CD163 ratio, with the median follow-up time of 65 months. This study protocol was approved by the ethics committees of Yantai Yuhuangding Hospital and Guangzhou Red Cross Hospital. The including criteria were as follows: (i) stage II-III patients with colon cancer or middle-high rectal cancer after radical resection (R_0_); (ii) availability of tissue specimens and follow-up data; (iii) without evidence of distant metastases and secondary primary cancers; and (iv) without preoperative anticancer therapy.

### Immunohistochemistry

Immunohistochemistry was performed on an automated platform (Benchmark-XT, Roche Company, Switzerland) according to the standard protocol provided by this platform. Primary antibodies against human CD86 (1:200, Affinity, USA) and CD163 (ready to use, Zhongshan Company, Beijing, China) were used to detect M1 and M2 macrophages, respectively. The Enzyme-labeled anti-mouse/rabbit polymerized secondary antibody (ready to use, Roche Company, Switzerland) was used for immunohistochemical staining.

### Evaluation of CD86+ or CD163+ Macrophages

According to the previous studies (22, 23), TAMs mainly infiltrated in the stromal area. So only TAMs that located in the stromal area were counted in our study. Each tissue section was evaluated under a high-power magnification field (HPF, 400×) using a Leica-DM-LB2 working station (Leica Microsystem, German). The intensity of CD86+ or CD163+ macrophages in tissue sections was determined according to the average number of CD86+ or CD163+ cells/HPF from three randomized fields in tumor stromal area. Two independently experienced pathologists evaluated the CD86 and CD163 staining intensity and were blinded to the clinical data, and the average values of three HPFs were used.

### Statistical Analysis

Statistical differences in clinical characteristics between low-ratio and high-ratio subgroups were determined by using the χ^2^ test. The main endpoints for this study were overall survival (OS) and recurrence-free survival (RFS). CRC patients were classified into the low-ratio and high-ratio subgroups according to the optimal cutoff point based on the Maxstat method (24). Survival differences between the high-ratio and low-ratio subgroups were assessed and compared by the Kaplan-Meier estimate and log-rank test. We also performed the multivariate Cox hazard regression analysis using the CD86/CD163 ratio and other clinical variables (age, sex, histological type, primary site, tumor stage, and adjutant chemotherapy) to calculate their hazard ratios (HRs) and 95% confidence intervals (CIs).

In addition, we performed the receiver operating characteristic (ROC) curve analysis to assess the predictive abilities of CD86/CD163 ratio and stage with the “pROC” package (25). To construct the ROC curves, CRC patients with a disease duration of ≤48 months were excluded if they had not experienced tumor recurrence at the last follow-up. The RFS times of the remaining patients were categorized into either ≤48 months or >48 months.

Using the training cohort, we constructed a prognostic nomogram based on the CD86/CD163 ratio and tumor stage with the “rms” package. We applied the calibration plot and concordance index (C-index) to examine its prediction abilities in two clinical cohorts (26, 27).

All analyses were performed by the R program (version 3.5.3). For all statistical analyses, p-values less than 0.05 were the criterion for statistical significance.

## Results

### The Relationship Between CD86/CD163 Ratio and Tumor Recurrence

#### Training Cohort

**Figure 1:**
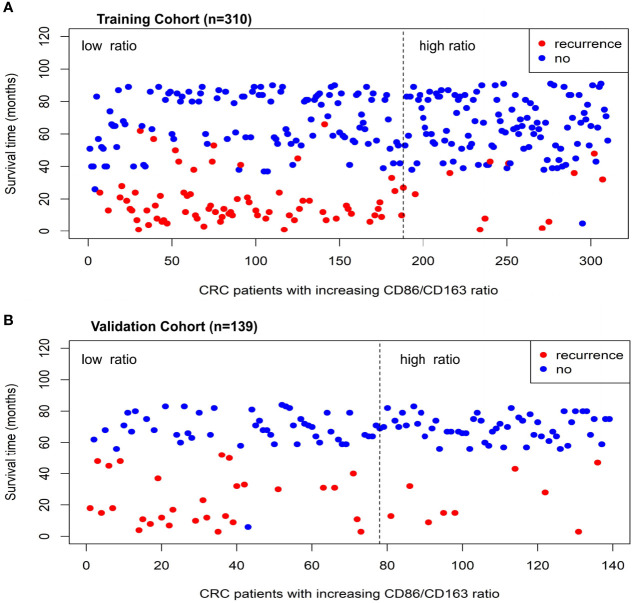
The relationship distributions of recurrence status and CD86/CD163 ratio in the training **(A)** and validation **(B)** cohorts.

The range of CD86/CD163 ratio in the training cohort is 0 to 7.09. Using the optimal cutoff point (0.51), CRC patients were stratified into the high-ratio (n=122) and low-ratio (n=188) subgroups. As the CD86/CD163 ratio increases, CRC patients potentially exhibit a decreasing incidence of tumor recurrence (**Figure 1A**).

#### Validation Cohort

The range of CD86/CD163 ratio in the validation cohort is 0 to 8.00. Using the same cutoff point (0.51), CRC patients were stratified into the high-ratio (n=61) and low-ratio (n=78) subgroups. As the CD86/CD163 ratio increases, CRC patients also potentially exhibit a decreasing incidence of tumor recurrence (**Figure 1B**).

### Survival Differences Between Different Ratio Subgroups

#### Training Cohort

**Figure 2:**
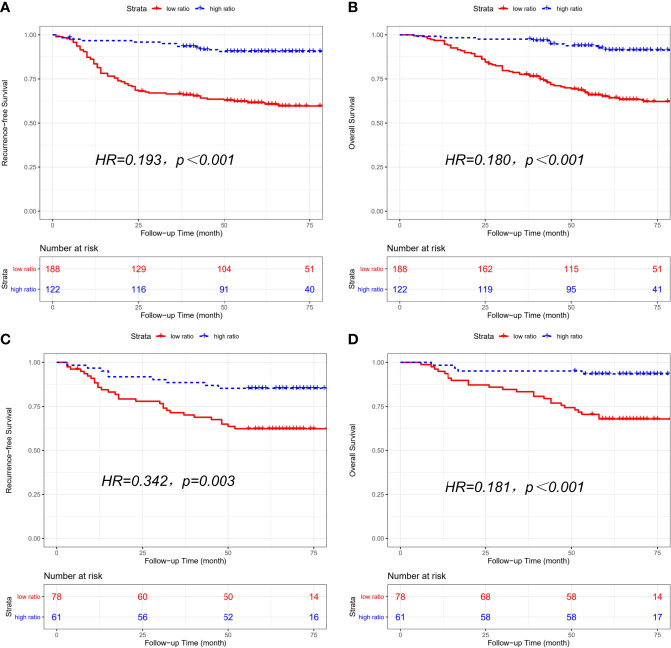
Kaplan-Meier curves of recurrence-free survival (RFS) and overall survival (OS) based on CD86/CD163 ratio for stage II-III CRC. Kaplan-Meier curves of RFS **(A)** and OS **(B)** in the training cohort. Kaplan-Meier curves of RFS **(C)** and OS **(D)** in the validation cohort.

Clinical characteristics of the high-ratio (n=122) and low-ratio (n=188) subgroups in the training cohort are shown in **Table 1**. Patients with a low CD86/CD163 ratio had shorter RFS (HR=0.193, 95% CI=0.102-0.364; *p*<0.001; **Figure 2A**) and OS (HR=0.180, 95% CI=0.090-0.362; *p*<0.001; **Figure 2B**) than patients with a high CD86/CD163 ratio in the training cohort.

**Table 1:** Clinical characteristics of CRC patients between low ratio and high ratio subgroups in the training and validation cohorts.

Variable	Training Cohort			Validation Cohort		
	low ratio	high ratio	*p*-value	low ratio	high ratio	*p*-value
**Age**						
<66 y	109	73	0.75	33	23	0.58
≥66 y	79	49		45	38	
**Gender**						
Male	121	71	0.27	38	32	0.66
Female	67	51		40	29	
**Histological Type**						
Non-mucinous cancer	169	112	0.57	66	55	0.33
Mucinous cancer	19	10		12	6	
**Primary Site**						
Colon	64	39	0.70	60	41	0.14
Rectum	124	83		18	20	
**Tumor Stage**						
II	70	69	<0.01	35	32	0.23
III	118	53		43	29	
**Adjutant Chemotherapy**						
Yes	132	77	0.19	48	37	0.92
No	56	45		30	24	

#### Validation Cohort

The clinical characteristics of the high-ratio (n=61) and low-ratio (n=78) CRC subgroups in the validation cohort are shown in **Table 1**. Patients with a low CD86/CD163 ratio had shorter RFS (HR=0.342, 95% CI=0.162-0.723; *p*=0.003; **Figure 2C**) and OS (HR=0.181, 95% CI=0.063-0.520; *p*<0.001; **Figure 2D**) than patients with a high CD86/CD163 ratio in the validation cohort.

### Multivariable Cox Regression Analysis

Multivariate Cox analysis revealed that CD86/CD163 ratio remained a powerful and independent prognostic factor for RFS (HR=0.233, 95% CI=0.123-0.443; *p*<0.001) and OS (HR=0.224, 95% CI=0.111-0.453; *p*<0.001) in the training cohort. We also obtained equivalent results for CD86/CD163 ratio in the validation cohort (**Table 2**).

**Table 2:** Multivariate Cox regression analysis of the CD86/CD163 ratio, clinical factors, and survival in the training and validation cohorts.

Variable	Recurrence-free Survival		Overall Survival	
	HR (95% CI)	*p*-value	HR (95% CI)	*p*-value
**Training Cohort (n=310)**				
CD86/CD163 Ratio (high ratio *vs.* low ratio)	0.233 (0.123-0.443)	<0.001	0.224 (0.111-0.453)	<0.001
Age (≥66 y *vs.* <66 y)	0.913 (0.567-1.468)	0.707	1.190 (0.722-1.962)	0.495
Gender (female *vs.* male)	1.113 (0.714-1.736)	0.637	0.992 (0.615-1.598)	0.972
Mucinous Cancer (yes *vs.* no)	1.198 (0.566-2.537)	0.637	1.055 (0.447-2.489)	0.903
Primary Locations (rectum *vs.* colon)	0.827 (0.508-1.348)	0.446	0.700 (0.425-1.153)	0.161
Tumor Stage (III *vs.* II)	2.959 (1.720-5.090)	<0.001	3.230 (1.799-5.799)	<0.001
Adjutant Chemotherapy (no *vs.* yes)	0.568 (0.325-0.991)	0.046	0.607 (0.343-1.078)	0.086
**Validation Cohort (n=139)**				
CD86/CD163 Ratio (high ratio *vs.* low ratio)	0.337 (0.159-0.717)	0.005	0.175 (0.060-0.506)	0.001
Age (≥66 y *vs.* <66 y)	1.971 (0.222-1.159)	0.107	3.404 (1.221-9.488)	0.019
Gender (female *vs.* male)	1.493 (0.775-2.876)	0.230	1.276 (0.605-2.691)	0.523
Mucinous Cancer (yes *vs.* no)	0.359 (0.085-1.507)	0.161	0.507 (0.118-2.176)	0.361
Primary Locations (rectum *vs.* colon)	0.600 (0.262-1.373)	0.227	0.629 (0.237-1.670)	0.352
Tumor Stage (III *vs.* II)	2.914 (1.410-6.020)	0.004	3.153 (1.340-7.418)	0.009
Adjutant Chemotherapy (no *vs.* yes)	1.529 (0.750-3.116)	0.243	1.420 (0.647-3.118)	0.382

HR, hazard ratio; CI, confidence index.

### ROC Curve Analysis

**Figure 3:**
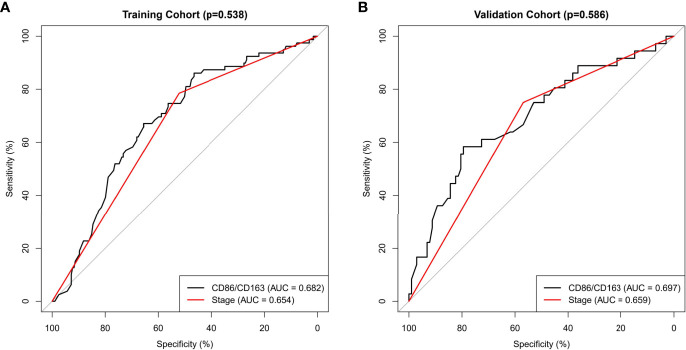
Receiver operating characteristic curves for CD86/CD163 ratio and tumor stage in the prediction of recurrence-free survival in the training **(A)** and validation **(B)** cohorts.

CD86/CD163 ratio might tend to have better predictive values than stage in the training (area under curve [AUC]: 0.682 *vs*. 0.654, *p*=0.538) and validation (AUC: 0.697 *vs*. 0.659, p=0.586) cohorts (**Figure 3**).

### Subgroup Analysis According to Tumor Stage and Chemotherapy Status

#### Tumor Stage

**Figure 4:**
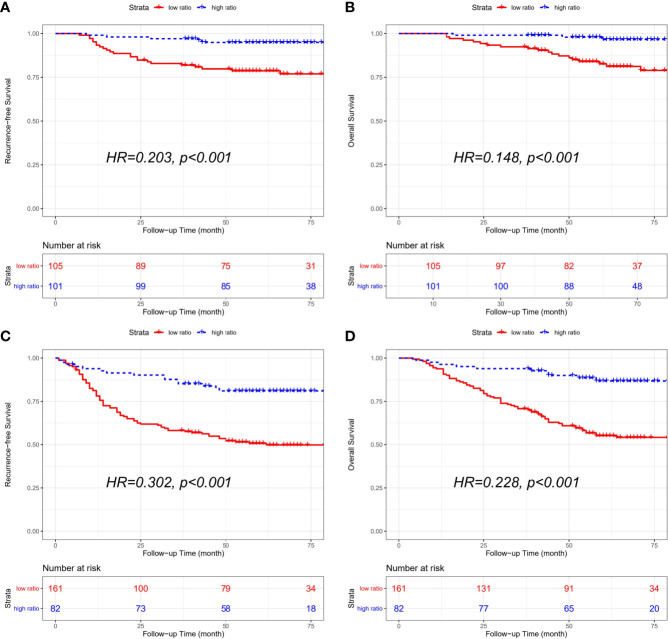
Kaplan-Meier curves of recurrence-free survival (RFS) and overall survival (OS) according to the CD86/CD163 ratio for stage II and stage III CRC alone in the combined cohort. Kaplan-Meier curves of RFS **(A)** and OS **(B)** in stage II CRC alone. Kaplan-Meier curves of RFS **(C)** and OS **(D)** in stage III CRC alone.

The combined cohort consisted of 206 stage II patients and 243 stage III patients. And stage II patients with a low CD86/CD163 ratio had shorter RFS (HR=0.203, 95% CI=0.077-0.534; p<0.001) and OS (HR=0.148, 95% CI=0.044-0.501; p<0.001) than stage II patients with a high CD86/CD163 ratio. We also obtained equivalent results for CD86/CD163 ratio in stage III CRC (**Figure 4**).

#### Chemotherapy Status

**Figure 5:**
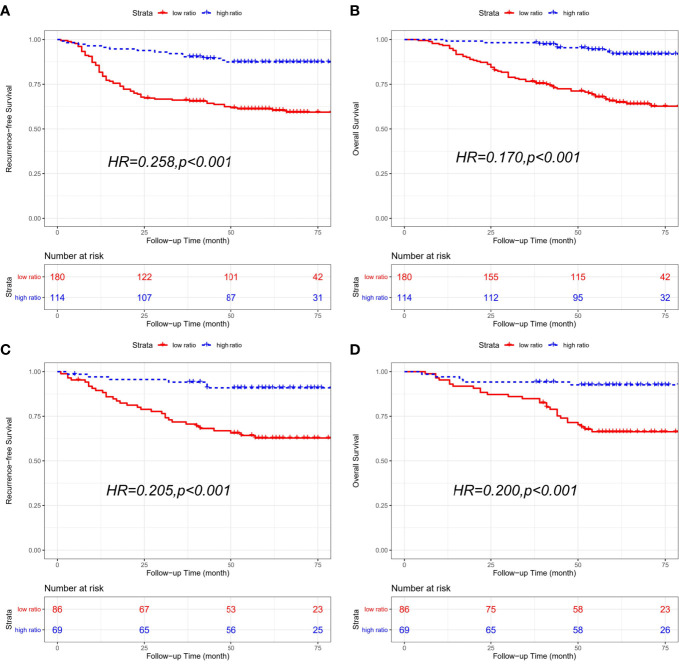
Kaplan-Meier curves of recurrence-free survival (RFS) and overall survival (OS) based on CD86/CD163 ratio for CRC patients with different chemotherapy statuses in the combined cohort. Kaplan-Meier curves of RFS **(A)** and OS **(B)** in CRC patients receiving adjutant chemotherapy. Kaplan-Meier curves of RFS **(C)** and OS **(D)** in CRC patients without receiving adjutant chemotherapy.

The combined cohort consisted of 294 patients with receiving chemotherapy and 155 patients without receiving chemotherapy. In CRC patients receiving adjutant chemotherapy, patients with a low CD86/CD163 ratio had shorter RFS (HR=0.258, 95% CI=0.145-0.458; p<0.001) and OS (HR=0.170, 95% CI=0.082-0.356; p<0.001) than patients with a high CD86/CD163 ratio. We also obtained equivalent results for CD86/CD163 ratio in CRC patients without receiving adjutant chemotherapy (**Figure 5**).

### Construction of a Prognostic Nomogram Based on CD86/CD163 Ratio and Stage

**Figure 6:**
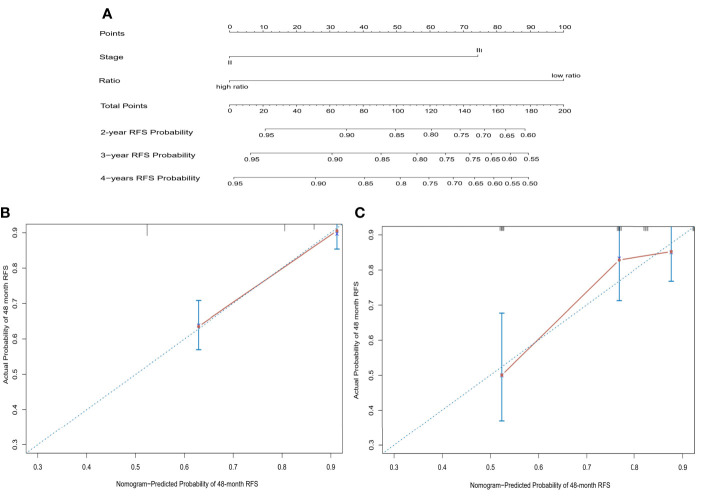
A prognostic nomogram based on CD86/CD163 ratio and tumor stage to predict the risk of tumor recurrence in stage II-III CRC **(A)** and further calibration curves of this nomogram to predict recurrence-free survival at 48 months in the training **(B)** and validation **(C)** cohorts.

**Figure 7:**
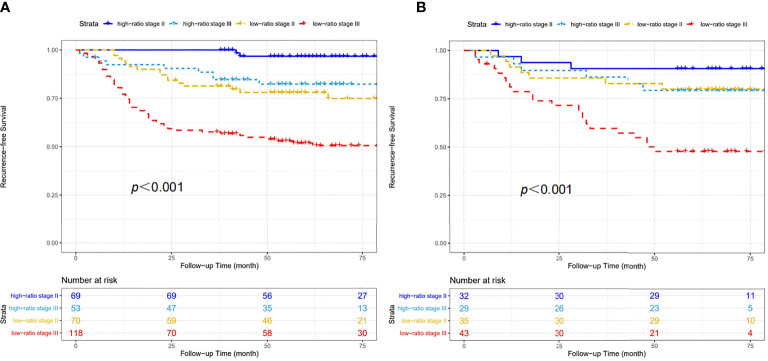
Four recurrence-risk subgroups according to the combination of CD86/CD163 ratio and tumor stage in the training **(A)** and validation **(B)** cohorts.

We constructed a prognostic nomogram based on CD86/CD163 ratio and stage in the training cohort (**Figure 6A**). The calibration plots for the 48-month RFS were predicted well in the training (C-index=0.732, **Figure 6B**) and validation (C-index=0.673, **Figure 6C**) cohorts. Furthermore, stage II-III patients were stratified into four recurrence-risk subgroups according to the combination of CD86/CD163 ratio and stage. Significant differences in RFS were noted among the four subgroups (low-ratio stage II, low-ratio stage III, high-ratio stage II, and high-ratio stage III) in the training (**Figure 7A**) and validation (**Figure 7B**) cohorts (all p<0.001).

## Discussion

By the immunohistochemistry staining, we constructed and validated a novel prognostic biomarker based on the stromal CD86/CD163 ratio to improve the prognostic stratification for stage II-III CRC. Our study demonstrated that CD86/CD163 ratio could effectively classify stage II-III patients with CRC into subgroups with high and low risks of postoperative recurrence. According to CD86/CD163 ratio and tumor stage, stage II-III patients were stratified into four recurrence-risk subgroups (high-ratio stage II, high-ratio stage III, low-ratio stage II, and low-ratio stage III). Of particular importance, this is the first study using standard immunohistochemical procedures that demonstrates the clinical utility of CD86/CD163 ratio as a postoperative prognostic tool in stage II-III CRC.

Adjutant chemotherapy after radical resection is recommended as the standard treatment strategy for stage III or high-risk stage II CRC (6–10). Previous evidence demonstrate that adjutant chemotherapy generally has limited benefits to stage II patients with CRC with an improved survival of 2-5% at 5 years after radical resection (10). In contrast, adjutant chemotherapy has shown robust efficacy in stage III CRC with an improved survival of 15-20% at 5 years (3–9). Six months of adjutant chemotherapy with oxaliplatin-based regimens has become the standard treatment for these patients. A significant proportion of CRC patients will experience cumulative neurotoxicity associated with oxaliplatin exposure. Grothey et al. (28) conducted a prospective pooled analysis and demonstrated that 3 months of adjutant treatment with either the CAPOX (oxaliplatin and capecitabine) or FOLFOX (oxaliplatin, fluorouracil, and leucovorin) regimen appeared to be sufficient in a lower-risk group (T1-3N1M0), especially when the CAPOX regimen was chosen. Six months of adjutant treatment with the CAPOX or FOLFOX regimen may be needed in a higher-risk group (T4N1M0/T1-4N2M0). Notably, our study demonstrated that tumor recurrence was heterogeneous even within the high-ratio and low-ratio subgroups of stage II-III CRC. Therefore, stage II-III CRC should be further stratified by CD86/CD163 ratio and stage into four recurrence-risk subgroups. This stratification may contribute to tailoring chemotherapy regimens and avoiding undertreatment or overtreatment in specific patients. We developed a prognostic nomogram based on CD86/CD163 ratio and tumor stage that allows for individualized estimation of the 48-month RFS probabilities among stage II-III CRC. Taken together with tumor stage, the stromal CD86/CD163 ratio may serve as a clinically useful tool to improve the prediction of tumor recurrence and guide more appropriate therapies for different risk subgroups.

The cross-network effect between the immune microenvironment and tumor cells may play critical roles in tumor occurrence and metastasis (11). The immune microenvironment may provide abundant resources for identifying novel recurrence biomarkers that allow for better stratification (11). Higher infiltration of CD3+ T cells, CD45RO+ memory T cells, and CD8+ cytotoxic T cells may be associated with decreased recurrence and mortality of CRC patients (29). Moreover, the quantitative analysis of two different lymphocyte subtypes (CD3/CD8, CD8/CD45RO, and CD3/CD45RO) revealed increased robustness and prognostic values for stages I-III CRC (30–32). These immune-based classifications are currently being introduced into the clinical settings for allowing the personal treatment.

Specifically, TAMs are one of the most represented lymphocyte subtypes in the immune microenvironment of solid tumors. TAMs are often separated into pro-inflammatory macrophages (M1-type) and anti-inflammatory macrophages (M2-type). Activated macrophages may be highly plastic immune cells and consist of a spectrum of activated states. And with M1-type and M2-type macrophages may only represent the extremes on two opposing ends of pro-inflammatory and anti-inflammatory states (18–20). M2 macrophages in tumor tissues was proved to promote angiogenesis and metastasis by secreting vascular endothelial growth factor (18, 33). Two previous studies involved only M2 TAMs and the total number of TAMs (M1+M2) instead of evaluating M1 and M2 TAMs separately (23, 34). In CRC patients, a high M2/(M1+M2) ratio may be significantly associated with increased recurrence and mortality of CRC patients (23, 34). This previous study implied that the infiltration of CD68+ TAMs wasn’t associated with recurrence and mortality of CRC, which may be attributed to the functional counterbalance modulated by the M1 and M2 TAMs (35). Although many studies have considered CD86 and CD163 as the cell surface markers of M1 and M2 TAMs, respectively, only a few studies have emphasized the clinical significance of CD86+ TAMs and CD163+ TAMs in solid tumors. High levels of CD86+ TAMs and low levels of CD163+ TAMs were closely related with a favorable prognosis in CRC patients (36, 37). And the polarization of M1 and M2 TAMs may be two ends of the macrophage spectrum. The combined analysis of CD86+ TAMs/CD163+ TAMs seems to be more appropriate for determining recurrence and mortality. In our study, high CD86/CD163 ratio subgroup hinted the polarization of M1 TAMs and served as a favorable prognostic factor for stage II-III CRC. On the other hand, low CD86/CD163 ratio subgroup exhibited the polarization of M2 TAMs and markedly correlated with decreased recurrence and mortality of CRC patients. These study results further emphasized the opposite functions of M1 and M2 TAMs. Until now, it was the first study about the ratio of M1/M2 (CD86+TAM/CD163+TAM) in stage II-III CRC.

But we should acknowledge some potential limitations for our study. First, our study design is retrospective in nature. So, this prognostic biomarker requires further validation in the prospective and multicenter cohorts before its clinical application. Secondly, our study is a real-world study. In such studies, some patients received adjuvant chemotherapy with the variant regimens, which may interfere with the study conclusions. In fact, the prognostic biomarkers must face such situations in the clinical practice. Thirdly, the detection method of TAMs needed the participation of experienced pathologists. The selection bias in the manual method couldn’t be completely avoided. However, the best way to solve this problem is to develop the automated method based on artificial intelligence.

In summary, our study demonstrated that CD86/CD163 ratio could effectively stratify stage II-III CRC into two different subgroups with a low and high risk of tumor recurrence and mortality. In addition, detection of CD86 and CD163 expression using the immunohistochemistry method is inexpensive and rapid and can be easily worked out in the pathology department of almost every hospital. Moreover, most patients could afford the cost associated with CD86 and CD163 detection for individual treatment. With further prospective studies of multiple-center cohorts, the CD86/CD163 ratio will aid in the personal treatment for stage II-III CRC.

## Data Availability Statement

The original contributions presented in the study are included in the article/supplementary material. Further inquiries can be directed to the corresponding authors.

## Ethics Statement

The studies involving human participants were reviewed and approved by Yantai Yuhuangding Hospital and Guangzhou Red Cross Hospital. The patients/participants provided their written informed consent to participate in this study. Written informed consent was obtained from the individual(s) for the publication of any potentially identifiable images or data included in this article.

## Author Contributions

GX, YM, and CJ conceived of and designed the study. GX, LJ, GQ, and ZL performed the experiments. GX, LJ, and ZL performed the data analysis. GX and JC reviewed this manuscript. All authors contributed to the article and approved the submitted version.

## Funding

This study protocol was supported by the High-level Talents Project of Liuzhou People Hospital (No. lrygcc202106), the Basic Research Ability Improvement Project of Young and Middle-aged Teachers from Guangxi colleges (No. 2021KY0111), and Self-funded project of Health Committee of Guangxi Zhuang Autonomous Region (Z20190901).

## Conflict of Interest

The authors declare that the research was conducted in the absence of any commercial or financial relationships that could be construed as a potential conflict of interest.

## Publisher’s Note

All claims expressed in this article are solely those of the authors and do not necessarily represent those of their affiliated organizations, or those of the publisher, the editors and the reviewers. Any product that may be evaluated in this article, or claim that may be made by its manufacturer, is not guaranteed or endorsed by the publisher.
